# Monocyte subsets display age-dependent alterations at fasting and undergo non-age-dependent changes following consumption of a meal

**DOI:** 10.1186/s12979-022-00297-6

**Published:** 2022-09-14

**Authors:** Ryan G. Snodgrass, Xiaowen Jiang, Charles B. Stephensen

**Affiliations:** 1 United States Department of Agriculture-Agricultural Research Services-Western Human Nutrition Research Center, Immunity and Disease Prevention Research Unit, Davis, CA USA; 2 Department of Nutrition, University of California Davis, Davis, CA USA

**Keywords:** Monocyte, Postprandial, Fasting, Ageing, Classical, Intermediate, Non-classical, Patrolling, Inflammation, Inflammaging

## Abstract

**Background:**

Monocytes are a heterogenous population of immune cells whose subsets and functions become substantially dysregulated with advanced age. Although much of our current understanding of the age-related changes in monocytes is derived from fasting blood samples, most people are predominately in the postprandial state during waking hours. As hormonal, metabolic, and immunological changes in response to the consumption of a meal are manifested in postprandial blood, it’s unclear how age-dependent changes in peripheral monocytes at fasting are impacted by a dietary challenge.

**Objective:**

We investigated the impact of age and meal consumption on circulating monocyte frequencies and subsets defined as classical (CD14 + CD16-), intermediate (CD14 + CD16 +), or non-classical (CD14dim CD16 +) in a cohort of 349 healthy adult volunteers grouped into categories based on their age: young adults (18–33 y, *n* = *123*), middle adults (34–49 y, *n* = *115*), and older adults (50–66 y, *n* = *111*).

**Results:**

Following 12-h fast total monocyte counts inversely correlated with subject age. Older adults had significantly fewer circulating monocytes along with elevated levels of TGs, cholesterol, glucose, IL-6, IL-8, TNF, neopterin, and CCL2 compared with young adults. Circulating monocyte pools in older adults consisted of smaller proportions of classical but larger proportions of intermediate and non-classical monocytes. Proportions of classical monocytes were inversely correlated with plasma TNF, IL-8, and neopterin while intermediate monocytes were positively correlated with plasma IL-6, TNF, and neopterin. Three hours after consuming a fat-containing meal postprandial monocyte counts increased in all age groups. Despite age-dependent differences in monocyte subsets at fasting, consumption of a meal induced similar changes in the proportions of classical and non-classical monocytes across age groups. Within the circulating postprandial monocyte pool, percentages of classical monocytes decreased while non-classical monocytes increased. However no change in precursory intermediate monocytes were detected. Our study confirms that ageing is associated with changes in monocyte frequencies and subsets and shows that consuming a fat-containing meal induces temporal changes in monocyte frequency and subsets independently of subject age.

**Clinical trial:**

Registered on ClincialTrials.gov (Identifier: NCT02367287)

**Supplementary Information:**

The online version contains supplementary material available at 10.1186/s12979-022-00297-6.

## Introduction

Monocytes are a heterogenous population of circulating blood cells which contribute to tissue integrity as well as to innate and adaptive immune defense [1]. In humans, there exist three well-characterized subsets based on their relative expression of surface antigens CD14 and CD16. Monocytes originate from myeloid precursors in the bone marrow and enter the circulation as classical monocytes (CLMs), identified as CD14 + CD16- [2]. CLMs, also called ‘inflammatory’ monocytes because they can extravasate into tissue to acutely contribute to the early inflammatory response, represent a transient cell population with a diverse differentiation potential [3]. In the steady state CLMs remain in circulation for approximately one day before either migrating into tissue to repopulate the tissue resident macrophage population or maturing into non-classical monocytes (NCMs) via the transitional intermediate monocyte (INM) population in circulation [2]. NCMs, identified as CD14dim CD16 + , have a much longer circulating lifespan of approximately 7 days and are referred to as ‘patrolling’ monocytes because they display a distinct motility and crawling pattern permitting them to actively surveil the endothelium and scavenge luminal debris while rarely extravasating into tissue [2, 4]. Although mechanisms driving the sequential transition from classical to NCM are not well understood, studies have shown that ageing greatly impacts the pool of circulating monocytes through modulating the subset maturation process [5–7].

Ageing coincides with alterations of immune function evident in poorer antigen-specific immune response and worse vaccine efficacy [8]. In addition to a decline in adaptive immunity, ageing presents with chronic, low-grade age-associated inflammation commonly referred to as “inflammaging” which contributes to the pathogenesis of many age-related diseases [9]. Inflammaging is associated with chronic activation of the innate immune system characterized by elevated levels of circulating pro-inflammatory cytokines including TNF, IL-6, and IL-8 [9]. In this respect, monocytes are considered an integral cell type in the etiology of inflammaging as the ageing pool of circulating monocytes is characterized by a shift towards increased NCMs displaying heightened basal pro-inflammatory cytokine production [10, 11].

Despite consistent observations of increased proportions of NCMs in the fasted state of older individuals, the extent to which intermediate and CLM subsets are also impacted by ageing is not currently resolved [7, 10–15]. In addition to age, a recent publication showed that both the frequency and functionality of circulating monocytes also differ between the fasted and postprandial state [16]. Considering that humans are predominantly in the postprandial state during waking hours, investigating the impact of ageing on monocyte subsets may need to consider the influence of the subject’s metabolic state. Therefore, to assess the impact of age as well as metabolic state on circulating monocytes we profiled monocyte frequency and subsets in healthy human volunteers both at fasting as well as after consumption of a mixed macronutrient challenge meal. We show that in the fasted state older volunteers (50–66 y) had reduced monocyte counts consisting of fewer CLMs but with increased intermediate and NCMs compared with young subjects (18–33 y). In response to consuming a high-fat meal, the postprandial monocyte pool expands and undergoes a dynamic age-independent shift towards increased proportions of NCMs without a concurrent rise in the precursory classical and intermediate subtypes. Collectively, our study confirms that ageing is strongly associated with changes in monocyte frequency and subsets and demonstrate that following consumption of a high-fat meal circulating monocytes undergo temporal changes in frequency and subsets independent of subject age.

## Methods

### Study participants and dietary challenge

Study participants were from the USDA Nutritional Phenotyping Study which included healthy men and women, aged 18–66 y with a normal to obese BMI of 18–44 kg/m^2^ living near Davis, California beginning in May 2015. Details of study recruitment and participation are contained in separate reports [17, 18]. Briefly, the study included two visits to the United States Department of Agriculture – Agricultural Research Service – Western Human Nutrition Research Center (WHNRC) scheduled within a period of 10–14 days. On visit 1 subjects were provided informed consent and screened to ensure the volunteers fell within designed ranges for the study. Visit 2 was the challenge meal test day. The night before visit 2, subjects were provided a high carbohydrate meal (17% kcal from fat, 77% kcal from carbohydrate, and 7.5% kcal from protein) and instructed to consume it before 19:00 h. Subjects arrived fasted (12 h) the next morning and fasting blood was collected before ingestion of a high-fat liquid challenge meal (60% kcal from fat, 25% kcal from carbohydrates, and 15% kcal from protein). Additional details of the standardized high carbohydrate dinner and high-fat challenge meal are contained in separate reports [19, 20]. Postprandial blood was drawn at 3 and 6 h after consumption of the challenge meal. Ethnicity was self-reported by subjects using a demographic questionnaire and is presented in Supplemental Table 1. Subjects were grouped White or Caucasian (*n* = 214), Hispanic or Latino/a (*n* = 45), Asian (*n* = 41), Multi-racial (*n* = 22), Black or African-American (*n* = 16), Middle Eastern (*n* = 5), Native Hawaiian or other Pacific Islander (*n* = 2), American Indian or Alaska Native (*n* = 1), or declined to respond (*n* = 3). The “Asian” group was comprised of subjects who responded as Asian, East Asian, South Asian, or Southeast Asian; Multi-racial subjects (“Multi”), identified as more than one ethnic group.

The study was registered at clinicaltrials.gov (identifier: NCT02367287) and received ethical approval from the University of California, Davis, Institutional Review Board. All participants provided written informed consent and received monetary compensation for their participation. Data were stored using the Research Electronic Data Capture (REDCap) application hosted by the University of California Davis Health System Clinical and Translational Science Center.

### Analysis of monocyte frequencies

Monocyte frequencies were quantified using complete blood count (CBC) with differential. During the four-year recruitment period from June 2015 through July 2019 the CBC analyses were performed using whole blood (treated with EDTA as an anticoagulant) in the UC Davis Health, Department of Pathology and Laboratory Medicine Clinical Laboratory using a Beckman Coulter LH750/780 (prior to October 2016) or a Beckman Coulter DxH800 automated hematology analyzer, with the exception that twelve samples early in the study (prior to August 14, 2015) were analyzed on an Abbott Cell-Dyn 322 analyzer at the WHNRC.

### Clinical parameters

Fasting and postprandial blood was collected and serum or plasma was obtained by centrifugation at 1300 × g at 4 °C for 10 min. Lipid-related markers including triglycerides, total cholesterol, HDL-cholesterol (HDL-C), and LDL-cholesterol (LDL-C) were measured using a Cobas Integra 400/800 kit (Roche, Indianapolis, IN), a Cobas CHOL2 kit (Roche), a Cobas HDL-C plus 3rd generation kit (Roche), and a Cobas LDLC3 kit (Roche), respectively. All assays were completed on an auto-analyzer, Cobas Integra 400 + instrument (Roche). Glucose concentrations in plasma samples were measured using Glucose HK Gen.3 kits (Roche) conducted on the Cobas Integra 400 + instrument (Roche). Serum insulin levels were determined by Elecsys Insulin kits running on a Cobas e411 analyzer (Roche).

### Plasma immune markers measured by ELISA

Neopterin concentration (nmol/L) was measured using undiluted sodium heparin plasma using a commercial, competitive enzyme immunoassay (Alpco, BRAHMS GmbH, Salem, NH, USA) according to the manufacturer’s instructions. Myeloperoxidase concentrations (µg/L) were measured using sodium heparin plasma (1:10 dilution) using a commercial ELISA kit (Alpco Immunodiagnostik, Salem, NH, USA) according to the manufacturer’s instructions. Soluble CD14 (sCD14) concentrations (µg/L) were measured in duplicate using sodium heparin plasma (1:400 or 1:600 dilution) using a commercial ELISA kit (Bio-Techne R&D Duoset Systems, Minneapolis, MN USA) according to the manufacturer’s instructions. Plates for these three assays were read on an Agilent BioTek Synergy reader (Santa Clara, CA USA) and data analyzed using the BioTek Gen5 software.

### Plasma immune markers measured by multiplexed MSD assay

The concentrations of plasma proteins (µg/L) were measured in plasma using MSD assay kits and the MSD sector imager 2400 (MESO Scale Discovery). EDTA plasma was used for proteins (CRP, SAA, ICAM-1, VCAM-1 using the Vplex Vascular Injury Panel 1 with samples diluted 1:1,000; CCL2 using the Vplex Custom Human Biomarker Chemokine Panel 1 with samples diluted 1:4; TNF, IL-1β, IL-6, IL-8 and IL-10 using the Vplex Custom Human Biomarker Proinflammatory Panel 1 with samples diluted 1:2). Three levels of lyophilized controls were used on each plate to assess plate-to-plate variation. Mean concentrations (µg/L) of duplicate wells were used for analysis.

### Analysis of monocyte subsets by flow cytometry

Monocyte subsets at fasting and postprandial time points were analyzed using 100 µl of whole blood collected into EDTA-treated tubes mixed with pre-titrated volumes of the following antibodies in BD Brilliant stain buffer (catalog # 563,794 BD Biosciences): CD45-BV786 (catalog # 563,716 BD Biosciences), CD91-PE (catalog # 550,497 BD Biosciences), CD14-BUV395 (catalog # 563,561 BD Biosciences), and CD16-BV421 (catalog # 562,878 BD Biosciences). Following a 20-min incubation at room temperature 1X BD FACS Lysing Solution (catalog# 349,202 BD Biosciences) was added to whole blood/antibody mixture and incubated at room temperature for an additional 10 min. Cells were washed twice with cold wash/stain buffer (containing 0.1% BSA (w/v), 0.05% NaN3 (w/v) in PBS) then analyzed using an LSR Fortessa flow cytometer (BD Biosciences) configured with blue (488 nm), red (640 nm), violet (405 nm) and UV lasers (355 nm). Data were collected using FACSDiva and analyzed using FlowJo version 10.6.1 software (BD Biosciences).

### Statistical analysis

All statistical analyses were performed in GraphPad Prism 9 (Version 9.3.1). Unless noted otherwise, graphical data are presented in violin plots as means with individual data points displayed. Data normality was assessed using the Anderson–Darling test. Cohort characteristics, experimental measurements, as well as monocyte frequencies and subsets at fasting were compared between age groups using Kruskal–Wallis non-parametric one-way ANOVA with Dunn’s multiple comparisons test. Paired monocyte frequencies and subsets were compared between metabolic state (fasting and at 3 and 6 h after meal consumption) using Friedman non-parametric, repeated measures one-way ANOVA with Dunn’s multiple comparisons test. Differences were considered significant when *P* < 0.05 (*P < 0.05; **P < 0.01; ***P < 0.001; ns = not significant). Correlation analysis was performed using non-parametric Spearman’s rank-order correlation. Correlations were considered significant when *P* < 0.05 (*P < 0.05; **P < 0.01; ***P < 0.001). Final assembly and preparation of all figures was done using CorelDRAW 2021 (Corel Corporation, Ottawa, Canada).

## Results

### Monocyte subsets in healthy adults differ with age

We studied circulating monocytes in a cohort of 349 healthy adult volunteers ranging from 18–66 y. Individuals were grouped into one of three categories based on their age: young adults (18–33 y), middle adults (34–49 y), and older adults (50–66 y). The ethnic composition of each group is presented in Supplemental Table 1. As intended by recruitment design, BMI was not different between age groups (Table 1). However, older adults had significantly higher fasting levels of triglycerides (TGs; *P* = 0.0024), total cholesterol (*P* < 0.0001), low-density lipoprotein (LDL) cholesterol (*P* < 0.0001), and glucose (*P* < 0.0001) compared to young adults. Additional descriptive characteristics and experimental measurements of the study cohort at fasting are presented in Table 1.

**Fig. 1:**
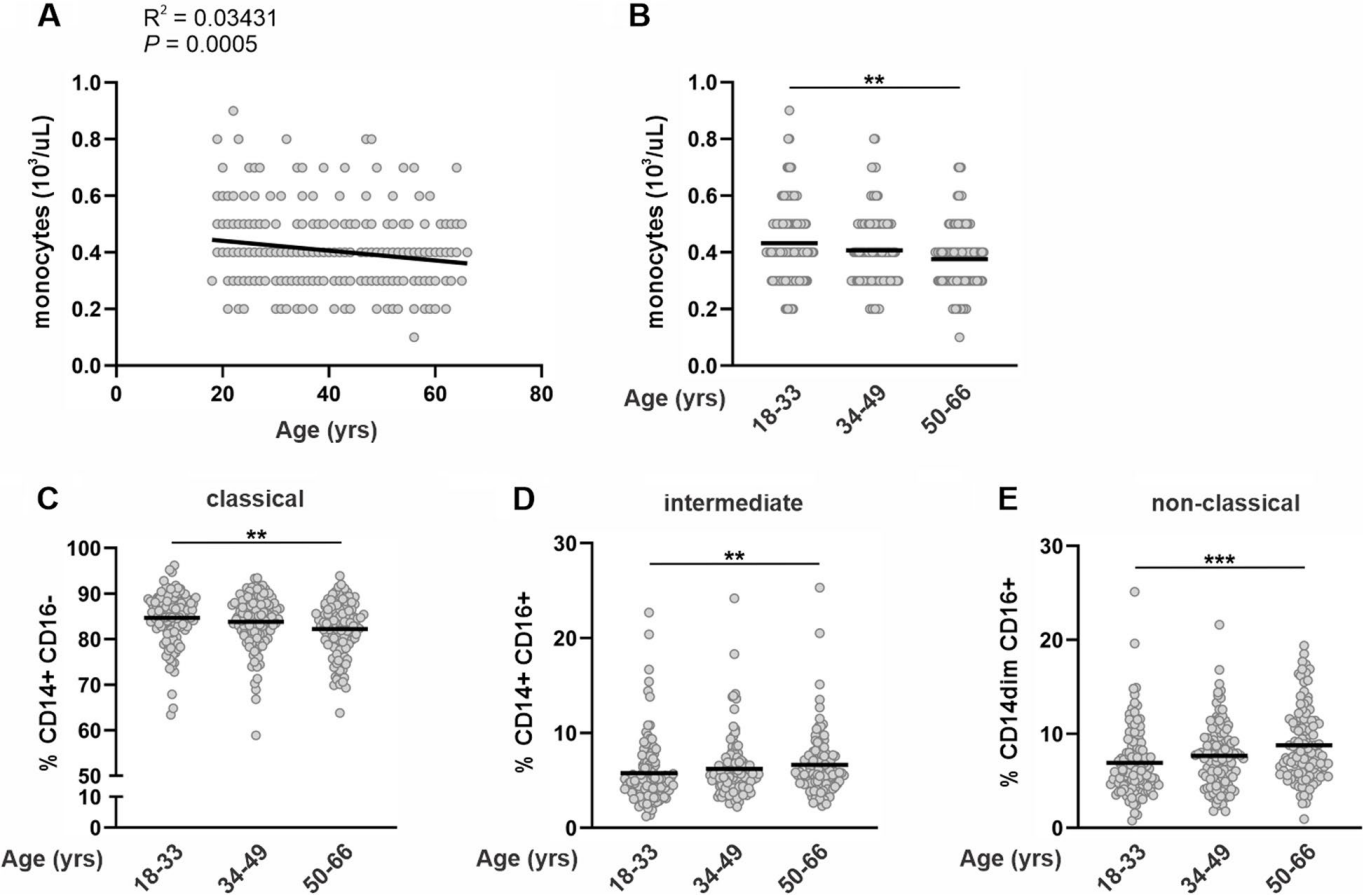
Fasting monocyte frequencies and phenotypes in study cohort of healthy adults. **A** Relation of absolute peripheral blood monocyte counts at fasting and subject age in years. Dark gray line indicates the linear line of best fit. Correlation squared (R^**2**^) and the *P* value for the correlation are shown. **B** Absolute monocyte numbers at fasting grouped by subject age. **C** Classical, **D** intermediate, and (**E**) non-classical monocytes displayed as percentage of total monocytes grouped by subject age. For B-E, mean and individual data points are shown. Statistical analyses were performed using Kruskal–Wallis non-parametric one-way ANOVA; *P < 0.05, **P < 0.01, ***P < 0.001

**Fig. 2:**
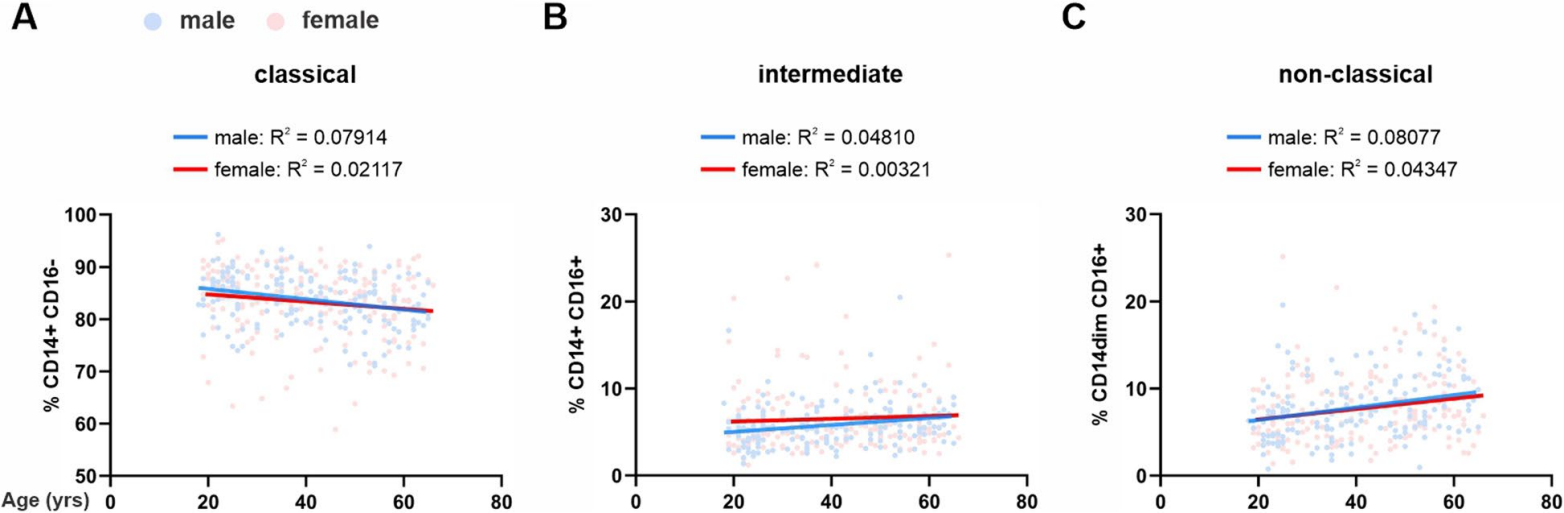
Monocyte subsets in fasting men and women of different ages. Relation of (**A**) classical, (**B**) intermediate, and (**C**) non-classical monocytes as a percentage of total monocytes and subject age in years. Blue and red lines indicate the linear line of best fit for male and female data points respectively. Correlation squared (R^**2**^) for the correlations are shown

Monocyte counts following a 12-h overnight fast inversely correlated with subject age **(**Fig. 1A**)**. Older adults had significantly fewer circulating monocytes compared with young adults (Fig. 1B and Table 1). To further assess age-related changes, circulating monocytes were identified by flow cytometry as classical (CD14 + CD16-), intermediate (CD14 + CD16 +), or non-classical (CD14dim CD16 +) subtypes according to expression of CD14 and CD16 on their surface as indicated in the gating strategy shown in Supplemental Fig. 1. CLMs comprised 84.7 ± 5.7% (mean ± SD) of circulating monocytes in young subjects but comprised a significantly smaller proportion in older subjects (82.2 ± 5.9%; *P* = 0.0020)). (Fig. 1C). In contrast to CLMs, intermediate and NCM which comprised 5.8 ± 3.4% and 6.9 ± 3.6% of circulating monocytes in young subjects respectively, comprised significantly larger proportions in older subjects (INMs: 6.6 ± 3.3%; *P* = 0.0023 and NCMs: 8.8 ± 3.9%; *P* = 0.0002) (Fig. 1D and E). Furthermore, linear regression analysis indicated that the slopes of the associations between subset and age for classical, intermediate, and NCMs were not significantly different between genders (Fig. 2A-C). Together these data show that ageing is associated with a smaller circulating monocyte pool composed of reduced proportions of CLMs but increased proportions of intermediate and NCMs.

### Correlation of soluble blood markers with fasting monocyte subsets

**Fig. 3:**
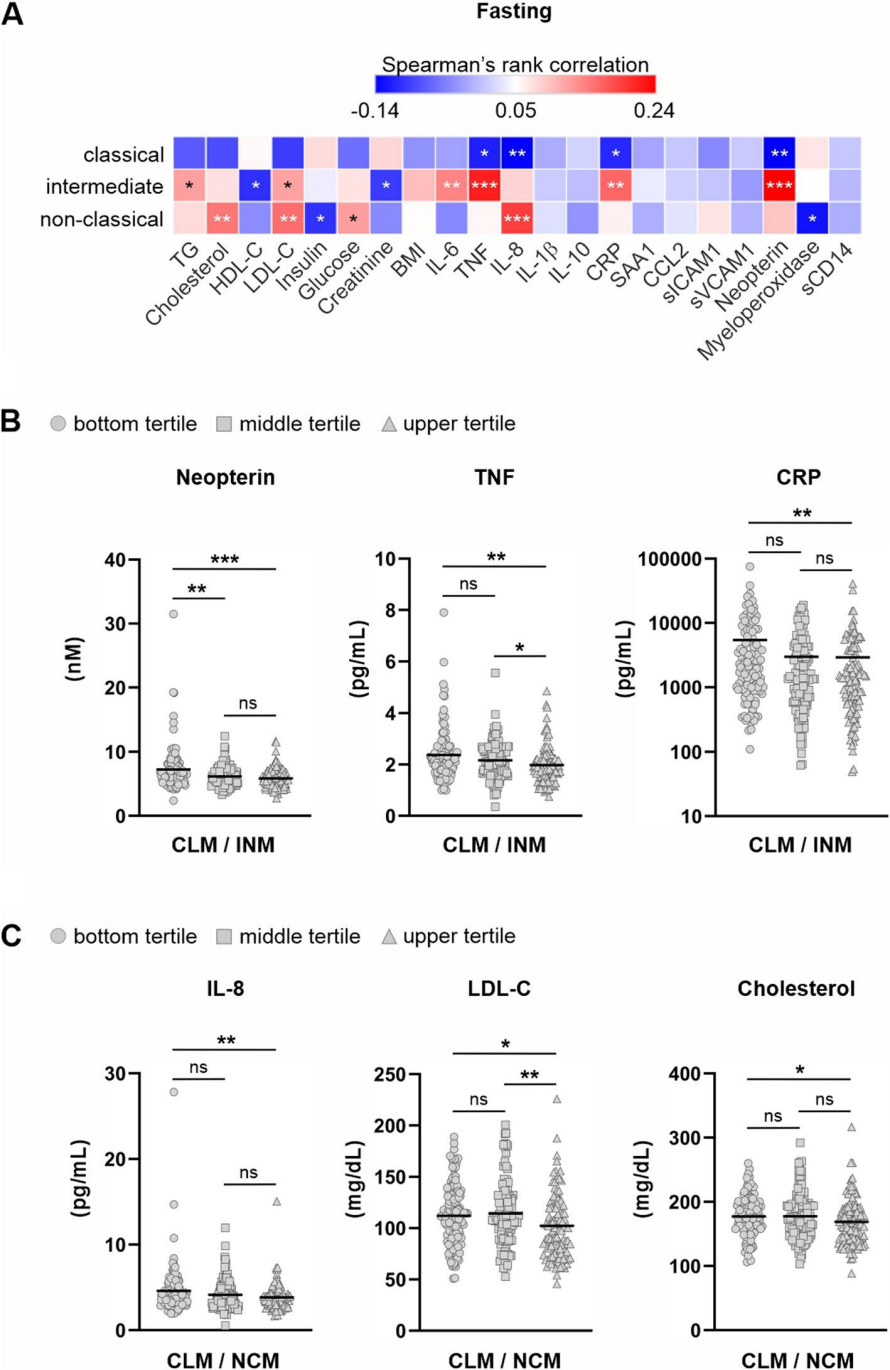
Correlations between fasting soluble factors and monocyte subsets. **A** Spearman’s rank correlations between fasting soluble factors and percent of classical, intermediate, and non-classical monocytes. Correlations were considered significant when *P* < 0.05 (*P < 0.05; **P < 0.01; ***P < 0.001). **B** Neopterin, TNF, and CRP levels in subjects grouped into tertiles based on the ratio of percent classical monocytes (CLMs) to percent intermediate monocytes (INMs) and, **C** IL-8, LDL-C, and cholesterol in subjects grouped into tertiles based on the ratio of percent classical monocytes (CLMs) to percent nonclassical monocytes (NCMs). Statistical analyses were performed using Kruskal–Wallis non-parametric one-way ANOVA; *P < 0.05, **P < 0.01, ***P < 0.001, ns = not significant

In addition to altered monocyte subsets and increased blood lipids in the fasted state, older subjects exhibited higher levels of pro-inflammatory cytokines TNF (*P* < 0.0001), IL-8 (*P* < 0.0001), and IL-6 (*P* = 0.0009) (Table 1 and Supplemental Fig. 2) compared to young subjects which is consistent with the “inflammaging” phenomenon described as a persistent, low-grade, chronic inflammation that occurs during ageing [9]. To investigate the relationship between soluble blood markers and monocyte subsets we performed non-parametric Spearman correlations. For percentages of CLMs, the strongest inverse associations were found for IL-8 (*ρ* = -0.1485; *P* = 0.0056) and neopterin (ρ = -0.1605; *P* = 0.0026) (Fig. 3A). For INM populations, IL-6 (*ρ* = 0.1393; *P* = 0.0093), TNF (ρ = 0.2164; *P* < 0.0001), CRP (*ρ* = 0.1614; *P* = 0.0026), and neopterin (*ρ* = 0.2425; *P* < 0.0001) displayed strong positive relationships while the percentage of NCMs displayed the strongest relationships with cholesterol (*ρ* = 0.1488; *P* = 0.0053), LDL-C (*ρ* = 0.1557; *P* = 0.0035), and IL-8 (*ρ* = 0.1920; *P* = 0.0003). As intermediate and NCMs are derived from precursory CLMs through sequential transition [2], we also assessed levels of soluble blood markers in subjects based on the rate of sequential transition from circulating CLMs. Subjects were grouped into tertiles based on their ratio of percent CLMs to percent INMs or NCMs. Subjects with CLM to INM ratios in the bottom tertile displayed significantly elevated levels of neopterin, TNF, and CRP compared to subjects in the upper tertile (Fig. 3B). Subjects with CLM to NCM ratios in the bottom tertile displayed significantly elevated levels of IL-8, LDL-C, and cholesterol compared to subjects in the upper tertile (Fig. 3C).

### Alterations of monocyte subsets after meal consumption is independent of age

**Fig. 4:**
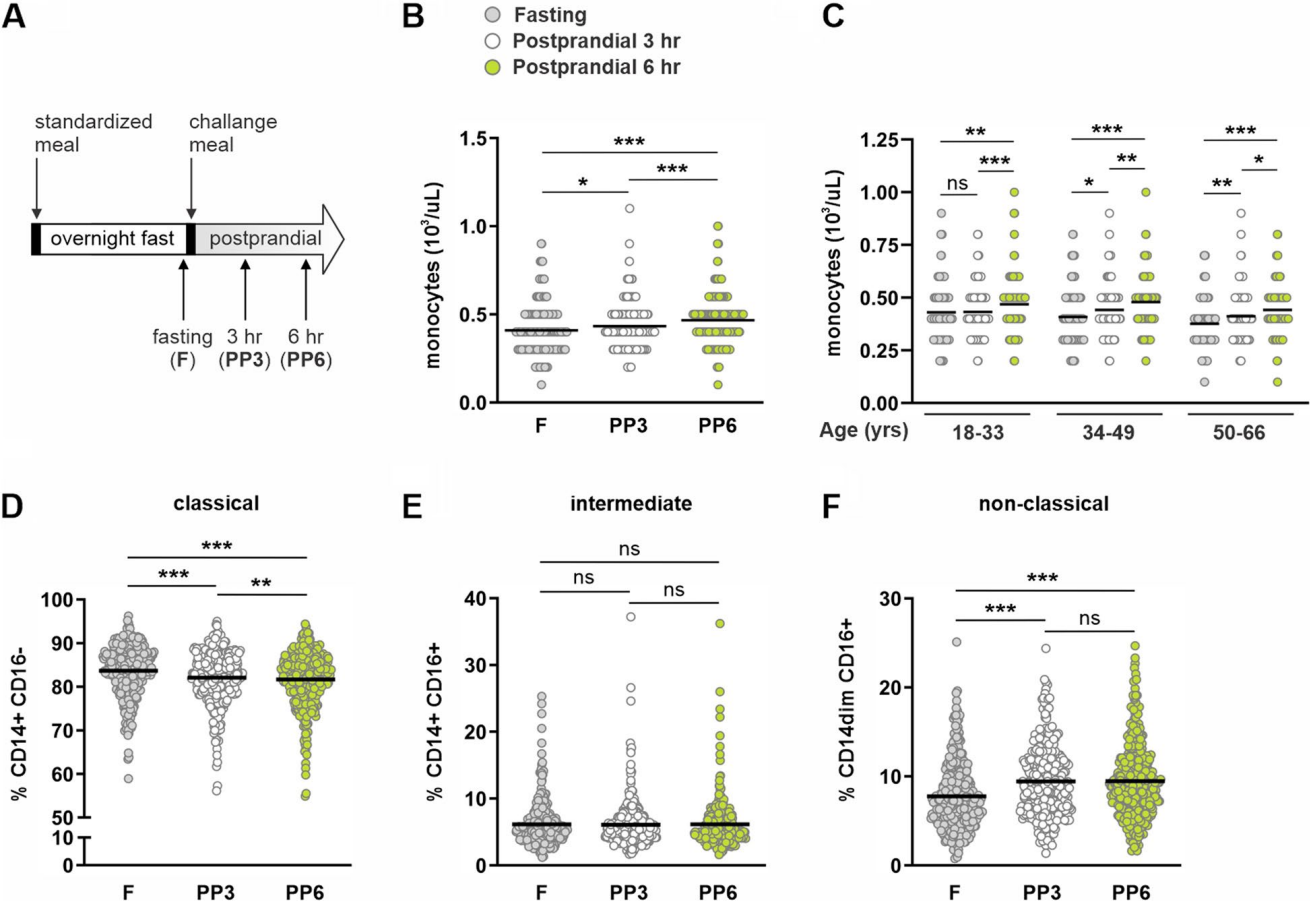
Fasting and postprandial monocyte counts and subsets. **A** Schematic representation of feeding regimen including standardized dinner meal and morning challenge meal with fasting and postprandial blood draws. Absolute numbers of peripheral blood monocytes at (**B**) fasting, 3, and 6 h after consumption of challenge meal for all study participants or (**C**) grouped by subject age. **D** classical, (**E**) intermediate, and (**F**) non-classical monocytes from all study subjects displayed as percentage of total monocytes. Mean and individual data points are shown. Statistical analyses in (**A**, **B**, **D**, **E**, and **F**) were performed using Friedman non-parametric, repeated measures one-way ANOVA; *P < 0.05, **P < 0.01, ***P < 0.001, ns = not significant. Statistical analysis in C was performed using Wilcoxon paired, non-parametric, two-tailed t test; ***P < 0.001, ns = not significant

After assessing the impact of age on circulating monocytes at fasting, we explored the effect of dietary intake by profiling circulating monocytes at 3 and 6 h after ingestion of a mixed macronutrient meal. A schematic of the study design is presented in Fig. 4A. To control for circadian and dietary variation, [19] and minimize the “second meal effect for plasma triacylglycerol release” shown by Robertson et al. to occur 12 h after consumption of a fat-containing meal [21] all subjects consumed a standardized high carbohydrate dinner meal 12 h before consuming a high-fat liquid challenge meal the following morning. Details of the standardized high carbohydrate dinner and high-fat challenge meal are contained in separate reports [19, 20]. In relation to baseline fasting conditions, postprandial monocyte counts were significantly elevated at 3 and 6 h after the meal (Fig. 4B), which are in line with previous reports in humans and mice [16, 22]. When stratified by subject age, all age-groups exhibited increased monocyte counts at 6 h after the meal but only middle and older adults displayed elevated monocyte counts at 3 h (Fig. 4C). Analysis of postprandial monocyte subsets revealed that CLMs, which constituted 83.7 ± 5.8% of circulating monocytes in all volunteers at baseline fasting conditions, decreased to 82.1 ± 6.0% (*P* < 0.0001) at 3 h and to 81.7 ± 6.2% (*P* < 0.0001) at 6 h after the meal (Fig. 4D). In a reciprocal manner, NCMs increased from 7.8 ± 3.7% of circulating monocytes at baseline fasting conditions to 9.4 ± 3.9% (*P* < 0.0001) at 3 h and to 9.5 ± 4.2% (*P* < 0.0001) at 6 h after the meal (Fig. 4F). Remarkably, no change in the percentage of INMs between fasting (6.1 ± 3.3%) and postprandial timepoints (3 h postprandial: 6.1 ± 3.4% and 6 h postprandial: 6.1 ± 3.4%) were observed (Fig. 4E).

**Fig. 5:**
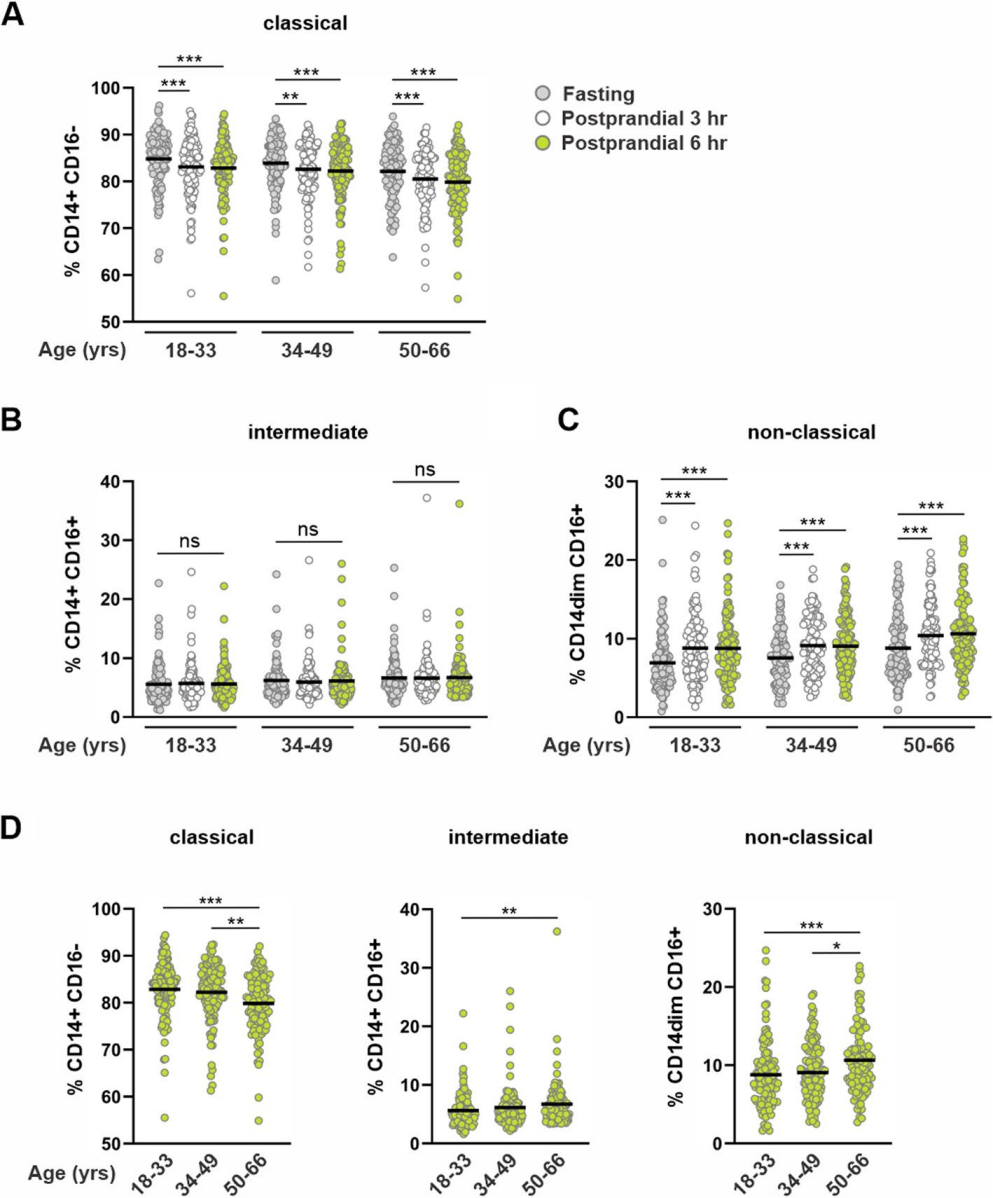
Fasting and postprandial monocyte subsets by subject age. Fasting, 3, and 6-h postprandial (**A**) classical, (**B**) intermediate, and (**C**) non-classical monocytes displayed as percentage of total monocytes and grouped by subject age. Six hour postprandial (**D**) classical, intermediate, and non-classical monocytes displayed as percentage of total monocytes and grouped by subject age. Mean and individual data points are shown. Within age-group analyses (**A**-**C**) were performed using Friedman non-parametric, repeated measures one-way ANOVA; *P < 0.05, **P < 0.01 ***P < 0.001, ns = not significant. Between age-group analyses (**D**) were performed using Kruskal–Wallis non-parametric one-way ANOVA; *P < 0.05, **P < 0.01, ***P < 0.001

Despite age-dependent differences in monocyte subsets at baseline fasting conditions, consumption of a high-fat meal induced a similar shift in the proportion of classical and NCMs in all age groups. At both 3 and 6 h after the meal, the proportion of CLMs decreased (Fig. 5A) while the proportion of NCMs increased in all age groups (Fig. 5C). Surprisingly, while the proportion of NCMs increased, no change in the proportion of the precursory INMs were detected in any age group (Fig. 5B). As the challenge meal induced an equivalent shift in the proportion of classical and NCMs, monocyte subsets at 6 h exhibited the same age-dependent profile as at baseline fasting conditions (Fig. 5D).

### Correlation of soluble blood markers with postprandial monocyte subsets

**Fig. 6:**
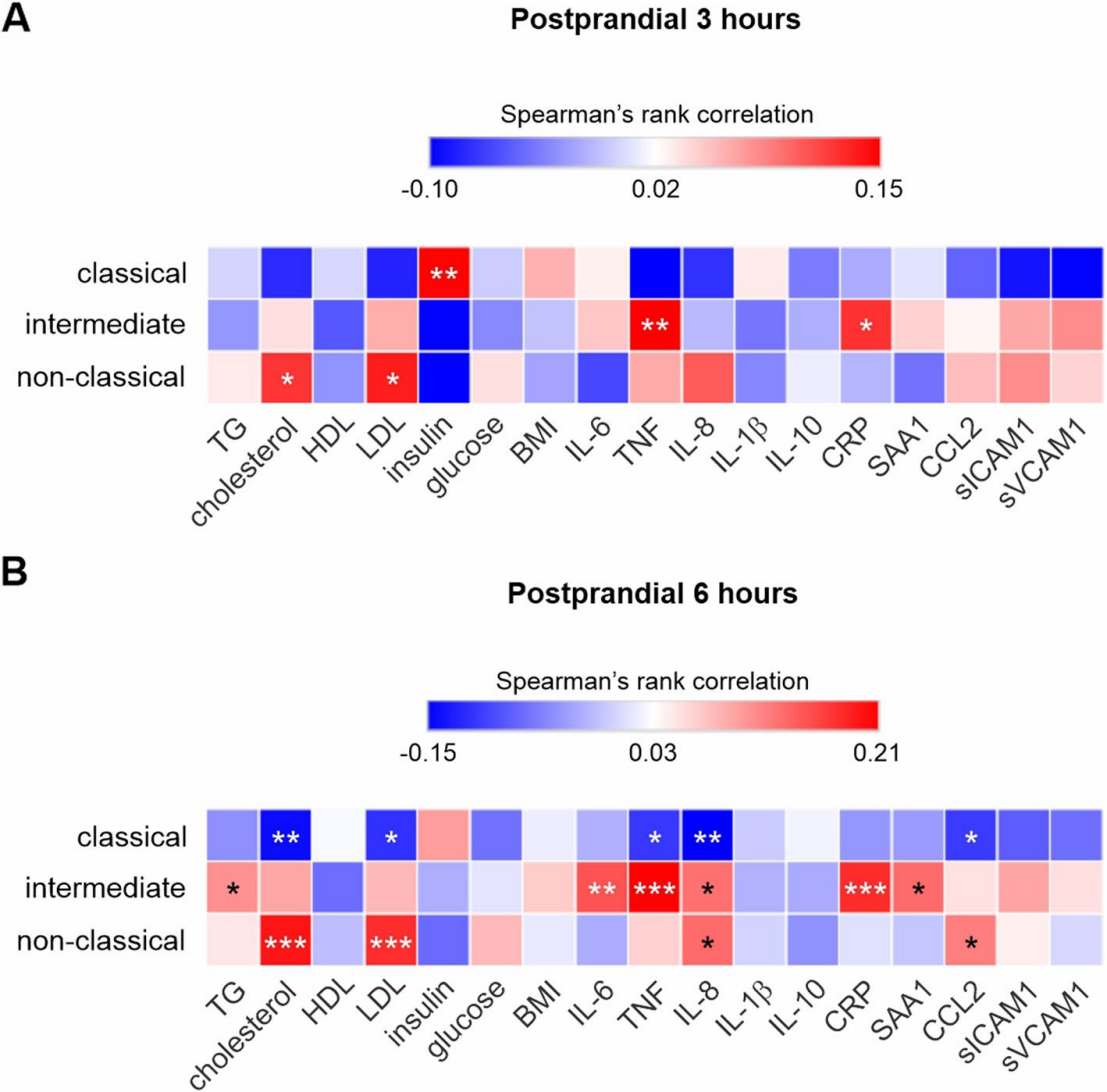
Correlations between postprandial soluble factors and monocyte subsets. Spearman’s rank correlations between 3 h (**A**) and 6 h (**B**) postprandial soluble factors and percent of classical, intermediate, and non-classical monocytes. Correlations were considered significant when *P* < 0.05 (*P < 0.05; **P < 0.01; ***P < 0.001)

To determine the relationship between soluble factors in postprandial blood with monocyte subset populations we tested their correlations at 3 and 6 h after meal. Although measured at baseline fasting, creatinine, neopterin, myeloperoxidase, and soluble CD14 were not measured in postprandial samples. Apart from insulin, which showed a positive association with CLMs (*ρ* = 0.1489; *P* = 0.0056), correlation coefficients at 3 h were smaller for each factor compared to those at baseline fasting conditions (Fig. 6A). Cholesterol (*ρ* = 0.1247; *P* = 0.0205 for NCMs), LDL (*ρ* = 0.1364; *P* = 0.0112 for NCMs), TNF (*ρ* = 0.1499; *P* = 0.0053 for INMs), and CRP (*ρ* = 0.1261; *P* = 0.0191 for INMs) all displayed significant positive relationships with intermediate and NCMs at 3 h. In contrast to the 3-h timepoint, correlations with monocyte subset populations at 6 h were higher for pro-inflammatory cytokines and more closely resembled correlations at baseline fasting conditions (Fig. 6B). TNF (*ρ* = -0.1113; *P* = 0.0403), IL-8 (*ρ* = -0.1503; *P* = 0.0056), cholesterol (*ρ* = -0.1419; *P* = 0.0088), LDL (*ρ* = -0.1153; *P* = 0.0336), and CCL2 (ρ = -0.1081; *P* = 0.0484) all showed significant inverse associations with CLM populations. TGs (*ρ* = 0.1074; *P* = 0.0481), IL-6 (*ρ* = 0.1514; *P* = 0.0052), TNF (*ρ* = 0.2096; *P* < 0.001), IL-8 (ρ = 0.1302; *P* = 0.0164), CRP (ρ = 0.1786; *P* = 0.0009), and SAA1 (ρ = 0.1348; *P* = 0.0129) exhibited significant positive relationships with INMs, while cholesterol (ρ = 0.1955; *P* = 0.0003), LDL (*ρ* = 0.1781; *P* = 0.0010), IL-8 (*ρ* = 0.1355; *P* = 0.0125), and CCL2 (*ρ* = 0.1211; *P* = 0.0269) all displayed significant positive relationships with NCM populations.

## Discussion

To investigate the impact of ageing on fasting and postprandial monocytes we examined monocyte frequencies and subsets in healthy adults recruited for a cross-sectional Nutritional Phenotyping Study [19]. In our study cohort of 349 individuals, we observed that total fasting monocyte counts were inversely associated with subject age and were significantly lower in older subjects compared to young subjects. Subset analysis based on the expression of CD14 and CD16 indicated that in relation to young adults, the proportion of CD14 + CD16- CLMs decreased, while CD14 + CD16 + intermediate and CD14dim CD16 + NCMs increased in older adults. Along with the observed changes in monocyte subsets, older adults also exhibited significantly increased plasma pro-inflammatory cytokines which supports the current paradigm that increased intermediate and NCMs contribute to chronic low-grade age-associated inflammation, or inflammaging [9, 11, 23]. Although intermediate and NCMs have been reported to increase with age [7, 10, 12], our observation of reduced proportions of CLMs in older subjects has been reported less often [6, 10].

What mediates the expansion of intermediate and NCMs in older adults is currently unknown. Analysis of myeloid cell kinetics in healthy rhesus macaques show that the half-life of blood circulating CLMs significantly declines with age which could lead to increased proportions of intermediate and NCMs due to their longer circulating lifespans [2, 24, 25]. Mechanistic studies investigating monocytes from young and old individuals have reported several functional alterations that may be responsible for the age-associated subset shift. Monocytes isolated from older adults also display impaired antigen presentation, phagocytosis, and bacterial clearance [7, 10]. Recently, NCMs were proposed as a senescent monocyte population based on observations that non-classical subsets exhibit shorter telomeres, increased expression of a senescence-associated microRNA, and produce increased levels of pro-inflammatory cytokines TNF, IL-6, and IL-8 in vitro [7, 10, 11]. Our results are very much in alignment with the current inflammaging paradigm as the oldest group of subjects in our cohort exhibited higher proportions of intermediate and NCMs along with elevated levels of TNF, IL-6, and IL-8. Although the extent to which intermediate and NCMs contribute to elevated levels of pro-inflammatory cytokines in vivo is not clear, we found significant positive correlations between circulating TNF, IL-6, and neopterin and the percentage of INMs as well as circulating IL-8 and the percentage of NCMs in our study cohort. In a recent report, a significantly high correlation between circulating neopterin and INM populations was also demonstrated [26]. Together, in vitro experiments showing intermediate and NCMs secrete higher levels of pro-inflammatory cytokines than do CLMs [11, 27] strongly support the paradigm that increased populations of intermediate and NCMs in older individuals contribute to increased chronic low-grade age-associated inflammation.

After investigating age-associated changes in monocyte subsets at fasting we assessed the impact of consuming a high-fat meal on monocyte frequencies and subtypes in volunteers of different ages. Our results showed that postprandial circulating monocytes increased at 3 and 6 h after meal consumption, which is well in agreement with Khan et al. who also observed increased levels of monocytes at 3 h after the consumption of a high-fat meal in a cohort of eleven lean subjects [22]. However, upon stratification of our postprandial monocyte data it became evident that in contrast to the 6-h timepoint, in which monocyte counts increased in all age-groups, only middle and older subjects exhibited elevated monocytes at 3 h after ingesting the high-fat meal. This discrepancy at 3 h may reflect the higher number of circulating monocytes at baseline fasting conditions in young adults. Although classical and NCM proportions differed between age groups at fasting, all age groups displayed a similar postprandial-mediated change in monocyte subsets following ingestion of the challenge meal exhibited by a reduced percentage of classical but increased percentage of NCMs. In comparison to the fasting state in which intermediate and NCM percentages increased concurrently with subject age, the postprandial state was marked by increased proportions of NCMs without a concurrent rise in INMs.

CLMs are released into the circulation from the bone marrow where they can undergo sequential transition to intermediate and non-classical subsets respectively [2]. While our age group-stratified data acquired at baseline fasting conditions support the current model of sequential transition as evident by increased proportions of intermediate and NCMs and decreased proportions of CLMs in older subjects, our postprandial data do not support the model. For one, at steady state monocytes enter the circulating pool as CLMs from bone marrow [2, 28]. However, in the postprandial state where total monocyte counts increased at 3 and 6 h after the meal relative to baseline fasting conditions, the percentage of CLMs within the circulating monocyte pool decreased. Second, despite increased NCM populations at 3 and 6 h after the meal, we were unable to detect a corresponding increase in precursory INMs. Taken together these results suggest that following consumption of a fat-containing meal monocytes may enter circulation, not as classical, but rather as NCMs. Alternatively, upon entering circulation in the postprandial state CLMs may bypass the sequential transition to intermediate and convert directly to NCMs.

Although functions of NCMs are still emerging, our data clearly demonstrate that their proportions increase in healthy adults following the consumption of a fat-containing meal in a manner that is independent of age. NCMs, also referred to as ‘patrolling monocytes’ because of their distinct motility and crawling pattern, contribute to vascular homeostasis and endothelial cell integrity by efficiently scavenging dying cells and luminal particles [4]. In our study cohort, circulating NCMs were strongly associated with concentrations of pro-atherogenic LDL-C and total cholesterol at fasting and at both postprandial time points. As the consumption of a high-fat meal is followed by increased circulating levels of TGs, cholesterol, and LDL and a profound reduction in endothelial function for up to 6 h [29], our data suggests that a temporal increase in NCMs may provide enhanced surveillance of endothelial integrity during the postprandial state.

In summary, our study comprised of healthy adult volunteers showed that the circulating monocyte pool undergoes significant alterations during ageing. In addition to confirming the expansion of the NCM subtype, we also show that older adults exhibit reduced monocyte counts comprised of fewer CLMs but with increased INMs. Our study also highlights a potential novel monocyte transition process in response to consuming a fat-containing meal as evident from increased postprandial monocyte counts comprised of increased proportions of NCMs without a concurrent rise in the precursory classical and intermediate subtypes.

**Table 1:** Characteristics and experimental measurements

		**Age Groups (years)**				
		**All Volunteers **	**18-33 **	**34-49 **	**50-66 **	**Significance **
	[n]	*349 *	*123 *	*115 *	*111 *	
Male/Female	[n]	*166/183 *	*59/64 *	*57/58 *	*50/61 *	
Age	[years]	40.3 (18-66)	22.8 (18-26)	33.4 (27-39)	46.5 (40-52)	
BMI	[kg/m2]	27.1 (18.0-43.3)	26.6 (18.2-40.0)	27.1 (18.0-43.3)	27.8 (18.3-42.2)	NS
total WBC	[103/uL]	5.4 (2.3-8.8)	5.9 (2.8-14.4)**a **	5.6 (2.5-9.4)**a **	5.2 (2.5-8.3)**b **	*P*<0.0001
total monocytes	[103/uL]	0.41 (0.1-0.9)	0.45 (0.2-0.9)**a **	0.41 (0.2-0.8)**ab **	0.39 (0.2-0.8)**b **	*P*=0.0027
TGs	[mg/dL]	95.8 (30-421)	83.4 (30-226)**a **	105.0 (36-421)b	99.8 (39-219)**b **	*P*=0.0004
Cholesterol	[mg/dL]	174.7 (88.5-316.8)	159.6 (88.5-261.6)**a **	175.7 (118.8-263.1)**b **	190.0 (108.6-316.8)**c **	*P*<0.0001
HDL-C	[mg/dL]	55.4 (23.8-116.0)	55.0 (27.5-109.4)	53.7 (23.8-108.3)	57.5 (27.9-116.0)	NS
LDL-C	[mg/dL]	109.8 (45.8-226.0)	96.5 (45.8-182.4)**a **	111.1 (65.8-200.8)**b **	123.0 (62.2-226.0)**c **	*P*<0.0001
Insulin	[mg/dL]	60.3 (9.6-284.2)	65.4 (12.0-252.2)	60.2 (12.0-284.2)	54.7 (9.6-245.3)	NS
Glucose	[mg/dL]	94.7 (62.4-322.6)	91.1 (62.4-139.5)**a **	96.4 (77.8-322.6)**b **	96.6 (78.1-138.2)**b **	*P*<0.0001
Creatinine	[μM]	68.5 (38.2-128.8)	68.9 (38.3-107.3)	69.1 (45.3-112.2)	67.3 (38.2-128.8)	NS
IL-6	[pg/mL]	0.70 (0.03-3.80)	0.61 (0.12-3.80)**a **	0.67 (0.03-2.38)**ab **	0.81 (0.24-3.02)**b **	*P*=0.0005
TNF	[pg/mL]	2.17 (0.35-7.90)	1.96 (0.35-5.55)**a **	2.14 (0.75-4.84)**ab **	2.43 (0.81-7.90)**b **	*P*<0.0001
IL-8	[pg/mL]	4.20 (0.54-27.81)	3.68 (1.78-14.68)**a **	3.93 (0.54-8.26)**a **	5.04 (1.97-27.81)**b **	*P*<0.0001
IL-1β	[pg/mL]	0.08 (0.00-1.18)	0.07 (0.00-1.18)**a **	0.08 (0.01-0.26)**b **	0.08 (0.00-0.30)**ab **	*P*=0.0122
IL-10	[pg/mL]	0.40 (0.08-23.86)	0.29 (0.08-2.17)**a **	0.39 (0.09-6.03)**b **	0.54 (0.11-23.86)**ab **	*P*=0.0331
CRP	[pg/mL]	3794 (50-75352)	3951 (62-75352)	3644 (50-40854)	3777 (54-32686)	NS
SAA1	[pg/mL]	9059 (184-252354)	10330 (263-252354)	6918 (184-135584)	9734 (245-213860)	NS
CCL2	[pg/mL]	100.7 (39.1-738.4)	94.3 (39.1-333.7)**a **	91.8 (44.6-216.6)**a **	116.8 (49.5-738.4)**b **	*P*<0.0001
Neopterin	[nM]	6.43 (2.39-31.48)	6.02 (2.74-19.25)**a **	6.45 (2.39-15.58)**b **	6.88 (3.86-31.48)**b **	*P*=0.0005
Myeloperoxidase	[ng/mL]	47.7 (9.4-412.9)	44.4 (9.4-385.6)	50.9 (11.1-412.9)	48.1 (18.7-303.9)	NS

**Abbreviations**: TGs, triglycerides; HDL-C, high-density lipoprotein cholesterol; LDL-C, low-density lipoprotein cholesterol; CRP, C-reactive protein; SAA1, serum amyloid A1; CCL2, C-C motif chemokine ligand 2.

The mean and range (in parenthesis) is given for quantitative parameters. Kruskal-Wallis non-parametric one-way ANOVA was used for evaluating significance and Dunn’s multiple comparisons test was used to compare groups. Means not sharing a common letter are significantly different. P<0.05 was considered significant. NS, not significant.

## Supplementary Information

**Additional file 1: Supplemental Figure 1. **Gating strategy for monocyte subset analysis by flow cytometry.

**Additional file 2: Supplemental Figure 2. **Fasting cytokines and experimental measurements. Fasting mean and individual data points for (**A**) IL-6, (**B**) TNF, (**C**) IL-8, (**D**) IL-1β, (**E**) IL-10, (**F**) CRP, (**G**) SAA1, (**H**) CCL2, (**I**) Neopterin, and (**J**) Myeloperoxidase grouped by subject age. Statistical analyses were performed using Kruskal-Wallis non-parametric one-way ANOVA with Dunn’s multiple comparisons test; *P<0.05, **P<0.01, ***P<0.001, ns = not significant.

**Additional file 3: Supplemental Table 1. **Ethnicities of study subjects.

## Data Availability

Requests for data from the USDA ARS WHNRC Nutritional Phenotyping Study used in this analysis should be made via an email to the senior WHNRC author on this publication. Requests will be reviewed quarterly by a committee consisting of the study investigators.

## Abbreviations

CLM: Classical monocyte
INM: Intermediate monocyte
NCM: Non-classical monocyte
CCL2: C–C Motif Chemokine Ligand 2
CD14: CD14 molecule
CD16: Fc Gamma Receptor IIIa
CRP: C-Reactive Protein
SAA1: Serum Amyloid A1

## References

[1] Guilliams M, Mildner A, Yona S (2018). Developmental and functional heterogeneity of monocytes. Immunity.

[2] Patel AA, Zhang Y, Fullerton JN, Boelen L, Rongvaux A, Maini AA (2017). The fate and lifespan of human monocyte subsets in steady state and systemic inflammation. J Exp Med.

[3] Robinson A, Han CZ, Glass CK, Pollard JW (2021). Monocyte Regulation in Homeostasis and Malignancy. Trends Immunol.

[4] Narasimhan PB, Marcovecchio P, Hamers AAJ, Hedrick CC (2019). Nonclassical Monocytes in Health and Disease. Annu Rev Immunol.

[5] Heimbeck I, Hofer TPJ, Eder C, Wright AK, Frankenberger M, Marei A (2010). Standardized single-platform assay for human monocyte subpopulations: Lower CD14+CD16++ monocytes in females. Cytom Part J Int Soc Anal Cytol.

[6] Verschoor CP, Johnstone J, Millar J, Parsons R, Lelic A, Loeb M (2014). Alterations to the frequency and function of peripheral blood monocytes and associations with chronic disease in the advanced-age, frail elderly. PLoS ONE.

[7] Seidler S, Zimmermann HW, Bartneck M, Trautwein C, Tacke F (2010). Age-dependent alterations of monocyte subsets and monocyte-related chemokine pathways in healthy adults. BMC Immunol.

[8] Mittelbrunn M, Kroemer G (2021). Hallmarks of T cell aging. Nat Immunol.

[9] Franceschi C, Garagnani P, Parini P, Giuliani C, Santoro A (2018). Inflammaging: a new immune-metabolic viewpoint for age-related diseases. Nat Rev Endocrinol.

[10] Hearps AC, Martin GE, Angelovich TA, Cheng WJ, Maisa A, Landay AL (2012). Aging is associated with chronic innate immune activation and dysregulation of monocyte phenotype and function. Aging Cell.

[11] Ong SM, Hadadi E, Dang TM, Yeap WH, Tan CTY, Ng TP (2018). The pro-inflammatory phenotype of the human non-classical monocyte subset is attributed to senescence. Cell Death Dis.

[12] Sadeghi HM, Schnelle JF, Thoma JK, Nishanian P, Fahey JL (1999). Phenotypic and functional characteristics of circulating monocytes of elderly persons. Exp Gerontol.

[13] Nyugen J, Agrawal S, Gollapudi S, Gupta S (2010). Impaired functions of peripheral blood monocyte subpopulations in aged humans. J Clin Immunol.

[14] Ault R, Dwivedi V, Koivisto E, Nagy J, Miller K, Nagendran K (2018). Altered monocyte phenotypes but not impaired peripheral T cell immunity may explain susceptibility of the elderly to develop tuberculosis. Exp Gerontol.

[15] Pence BD, Yarbro JR (2018). Aging impairs mitochondrial respiratory capacity in classical monocytes. Exp Gerontol.

[16] Jordan S, Tung N, Casanova-Acebes M, Chang C, Cantoni C, Zhang D (2019). Dietary intake regulates the circulating inflammatory monocyte pool. Cell.

[17] Bouzid YY, Arsenault JE, Bonnel EL, Cervantes E, Kan A, Keim NL (2021). Effect of Manual Data Cleaning on Nutrient Intakes Using the Automated Self-Administered 24-Hour Dietary Assessment Tool (ASA24). Curr Dev Nutr.

[18] Dimitratos SM, Hercules M, Stephensen CB, Cervantes E, Laugero KD (2021). Association between physiological stress load and diet quality patterns differs between male and female adults. Physiol Behav.

[19] Baldiviez LM, Keim NL, Laugero KD, Hwang DH, Huang L, Woodhouse LR (2017). Design and implementation of a cross-sectional nutritional phenotyping study in healthy US adults. BMC Nutr.

[20] Newman JW, Krishnan S, Borkowski K, Adams SH, Stephensen CB, Keim NL. Assessing Insulin Sensitivity and Postprandial Triglyceridemic Response Phenotypes With a Mixed Macronutrient Tolerance Test. Front Nutr. 2022;9:877696. 10.3389/fnut.2022.877696. 10.3389/fnut.2022.877696 PMC9131925 35634390

[21] Robertson MD, Henderson RA, Vist GE, Rumsey RDE (2002). Extended effects of evening meal carbohydrate-to-fat ratio on fasting and postprandial substrate metabolism. Am J Clin Nutr.

[22] Khan IM, Pokharel Y, Dadu RT, Lewis DE, Hoogeveen RC, Wu H (2016). Postprandial monocyte activation in individuals with metabolic syndrome. J Clin Endocrinol Metab.

[23] De Maeyer RPH, Chambers ES (2021). The impact of ageing on monocytes and macrophages. Immunol Lett.

[24] He Z, Allers C, Sugimoto C, Ahmed N, Fujioka H, Kim WK (2018). Rapid turnover and high production rate of myeloid cells in adult rhesus macaques with compensations during aging. J Immunol Baltim Md 1950.

[25] Sugimoto C, Hasegawa A, Saito Y, Fukuyo Y, Chiu KB, Cai Y (2015). Differentiation Kinetics of Blood Monocytes and Dendritic Cells in Macaques: Insights to Understanding Human Myeloid Cell Development. J Immunol Baltim Md 1950.

[26] Gjelstrup MC, Stilund M, Petersen T, Møller HJ, Petersen EL, Christensen T (2018). Subsets of activated monocytes and markers of inflammation in incipient and progressed multiple sclerosis. Immunol Cell Biol.

[27] Patel VK, Williams H, Li SCH, Fletcher JP, Medbury HJ (2017). Monocyte inflammatory profile is specific for individuals and associated with altered blood lipid levels. Atherosclerosis.

[28] Trzebanski S, Jung S (2020). Plasticity of monocyte development and monocyte fates. Immunol Lett.

[29] Thom NJ, Early AR, Hunt BE, Harris RA, Herring MP (2016). Eating and arterial endothelial function: a meta-analysis of the acute effects of meal consumption on flow-mediated dilation. Obes Rev Off J Int Assoc Study Obes.

