# Supplementary figures and images for: Monocyte subsets display age-dependent alterations at fasting and undergo non-age-dependent changes following consumption of a meal
Source: Immun Ageing. 2022 Sep 14;19:41. doi: 10.1186/s12979-022-00297-6 (PMC9472410; doi:10.1186/s12979-022-00297-6)

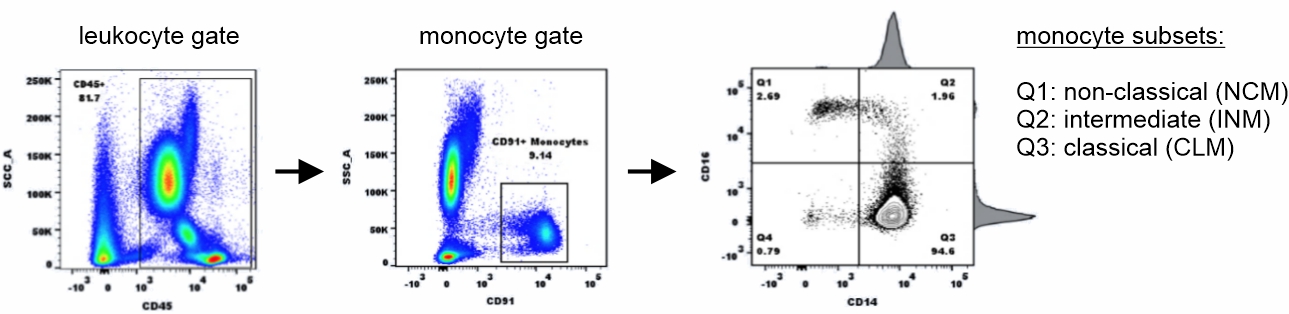

Supplement: Supplementary file 1 — Additional file 1: Supplemental Figure 1. Gating strategy for monocyte subset analysis by flow cytometry. [file 12979_2022_297_MOESM1_ESM.jpg]

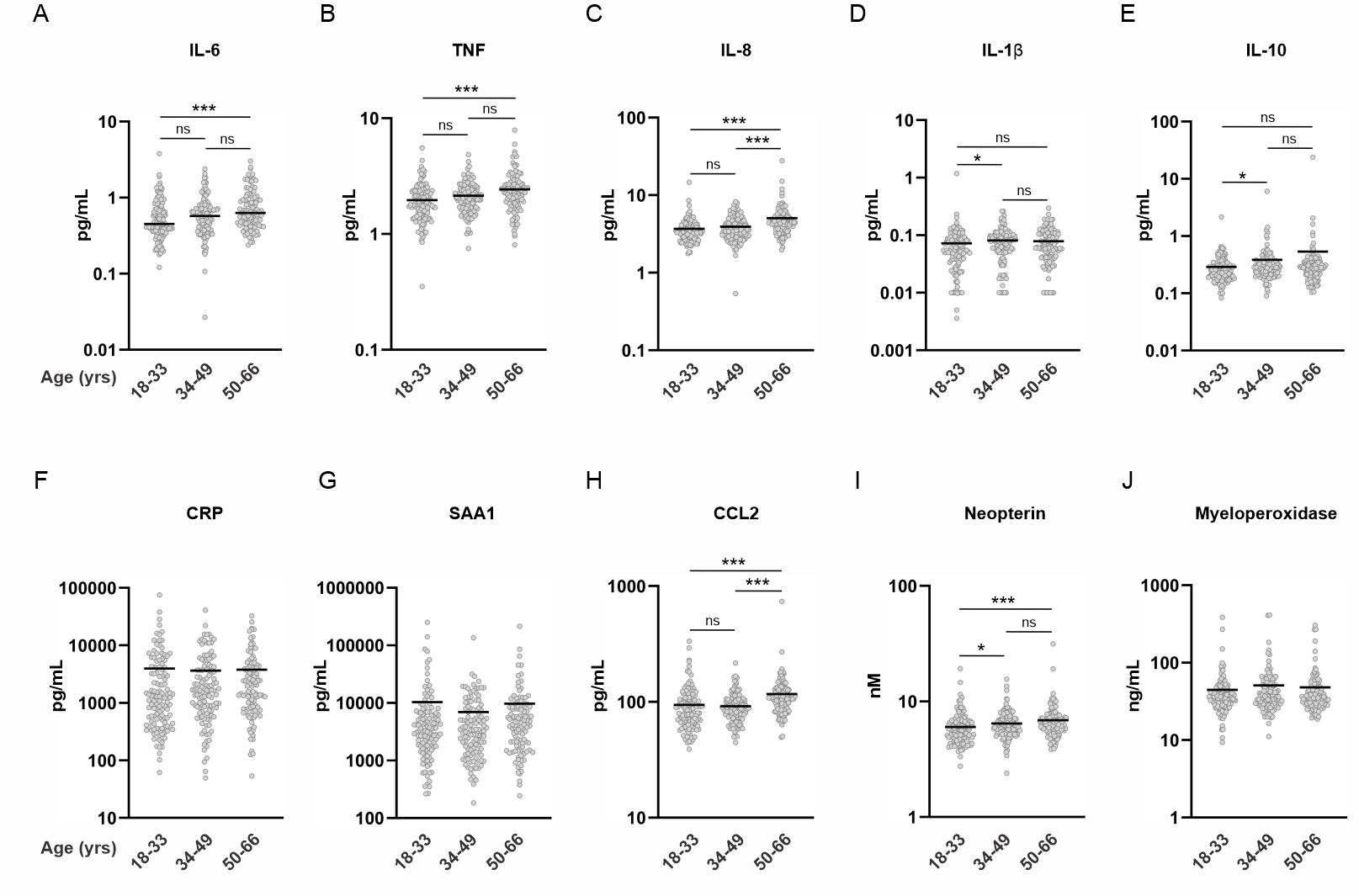

Supplement: Supplementary file 2 — Additional file 2: Supplemental Figure 2. Fasting cytokines and experimental measurements. Fasting mean and individual data points for (A) IL-6, (B) TNF, (C) IL-8, (D) IL-1β, (E) IL-10, (F) CRP, (G) SAA1, (H) CCL2, (I) Neopterin, and (J) Myeloperoxidase grouped by subject age. Statistical analyses were performed using Kruskal-Wallis non-parametric one-way ANOVA with Dunn’s multiple comparisons test; *P<0.05, **P<0.01, ***P<0.001, ns = not significant. [file 12979_2022_297_MOESM2_ESM.jpg]
