# Supplementary material for: Monocyte subsets display age-dependent alterations at fasting and undergo non-age-dependent changes following consumption of a meal
Source: Immun Ageing. 2022 Sep 14;19:41. doi: 10.1186/s12979-022-00297-6 (PMC9472410; doi:10.1186/s12979-022-00297-6)
Supplement: Supplementary file 3 — Additional file 3: Supplemental Table 1. Ethnicities of study subjects. [file 12979_2022_297_MOESM3_ESM.pdf]

Supplemental Table 1. Ethnicities of study subjects

Age Groups (years)					
18-33		34-49		50-66	
n = 123		*n = 115*		*n = 111*	
Asian:	25	Asian:	9	Asian:	7
Black or African-American:	9	Black or African-American:	3	Black or African-American:	4
Hispanic or Latino/a:	22	Hispanic or Latino/a:	12	Hispanic or Latino/a:	11
Middle Eastern:	4	Middle Eastern:	0	Middle Eastern:	1
American Indian or Alaska Native:	0	American Indian or Alaska Native:	0	American Indian or Alaska Native:	1
Multi:	10	Multi:	7	Multi:	5
Native Hawaiian or other Pacific Islander:	0	Native Hawaiian or other Pacific Islander:	1	Native Hawaiian or other Pacific Islander:	1
White or Caucasian:	52	White or Caucasian:	83	White or Caucasian:	79
Decline to respond:	1	Decline to respond:	0	Decline to respond:	2

The “Asian” group is comprised of subjects who responded as Asian, East Asian, South Asian, or Southeast Asian; Multi-racial subjects (“Multi”), identified as more than one ethnic group.
